# Biowaste-Derived Carbon Dots: A Perspective on Biomedical Potentials

**DOI:** 10.3390/molecules27196186

**Published:** 2022-09-21

**Authors:** Navid Rabiee, Siavash Iravani, Rajender S. Varma

**Affiliations:** 1 School of Engineering, Macquarie University, Sydney, NSW 2109, Australia; 2 Department of Materials Science and Engineering, Pohang University of Science and Technology (POSTECH), 77 Cheongam-ro, Nam-gu, Pohang 37673, Gyeongbuk, Korea; 3 Faculty of Pharmacy and Pharmaceutical Sciences, Isfahan University of Medical Sciences, 81746-73461 Isfahan, Iran; 4 Regional Centre of Advanced Technologies and Materials, Czech Advanced Technology and Research Institute, Palacký University in Olomouc, Šlechtitelů 27, 783 71 Olomouc, Czech Republic

**Keywords:** biowaste-derived carbon dots, green chemistry, sustainability, valorization, biocompatibility, biomedical applications

## Abstract

Today, sustainable and natural resources including biowastes have been considered attractive starting materials for the fabrication of biocompatible and biodegradable carbon dots (CDs) due to the benefits of availability, low cost, biorenewability, and environmentally benign attributes. These carbonaceous nanomaterials have been widely explored in the field of sensing/imaging, optoelectronics, photocatalysis, drug/gene delivery, tissue engineering, regenerative medicine, and cancer theranostics. Designing multifunctional biowaste-derived CDs with a high efficacy-to-toxicity ratio for sustained and targeted drug delivery, along with imaging potentials, opens a new window of opportunity toward theranostic applications. However, crucial challenges regarding the absorption/emission wavelength, up-conversion emission/multiphoton fluorescence mechanisms, and phosphorescence of these CDs still need to be addressed to attain the maximum functionality and efficacy. Future studies ought to focus on optimizing the synthesis techniques/conditions, evaluating the influence of nucleation/growth process on structures/properties, controlling their morphology/size, and finding the photoluminescence mechanisms. Reproducibility of synthesis techniques is another critically important factor that needs to be addressed in the future. Herein, the recent developments related to the biowaste-derived CDs with respect to their biomedical applications are deliberated, focusing on important challenges and future perspectives.

## 1. Introduction

**Figure 1:**
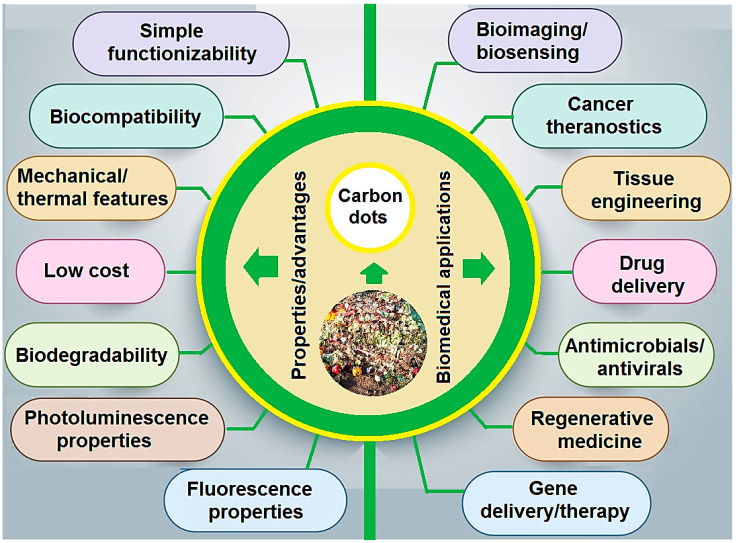
Advantages/properties and biomedical applications of biowaste-derived CDs.

Biowastes have been broadly utilized as natural and sustainable resources for the synthesis of various nanomaterials with biomedical potential [1,2,3,4]. However, in-depth analyses regarding scalability issues, extraction yields, optimization/purification processes, public accessibility, and biosafety are still required. The identification of best valorization and synthesis techniques for biowaste-derived nanomaterials is an important and challenging issue, due to extreme heterogeneity in biowastes [5,6,7]. In this context, carbon dots (CDs) with sizes of less than 10 nm exhibited significant advantages such as high biocompatibility, unique mechanical/thermal features, simple functionality, and multifunctionality properties [5,8]. CDs with strong fluorescence and unique photoluminescence/electrochemical properties compared with traditional fluorescent dyes (e.g., fluorescence organic agents and semiconductor quantum dots) illustrated excellent fluorescence properties such as photostability, resistance to photo-bleaching, and non-blinking [9]. In addition, they have shown better biocompatibility over inorganic nanomaterials such as CdSe or PbS quantum dots [10]. In view of all these unique properties, CDs have found wide clinical and biomedical applications, especially for bioimaging/biosensing, cancer theranostics, tissue engineering, regenerative medicine, drug/gene delivery, and antimicrobials/antivirals, among others (Figure 1) [11]. CDs may possess chemical functional groups such as polymer chains, or oxygen- and amino-based groups on their surfaces, which play crucial roles in their photoluminescence behavior [12]. The surface functionalization of CDs can play vital functions in improving their properties, thus making them potential candidates in the fields of bio- and nano-medicine [11]. Notably, optimized and controllable fabrication and modification procedures still need to be further evaluated for manufacturing CDs with well-defined sizes and morphologies as well as the desired efficacy and functionality. Considering the scalable and sustainable synthesis of CDs, natural renewable biowastes can be used for developing materials with specific physicochemical properties suitable for biomedical applications [11,13,14].

The reason for using leaf extracts and naturally extracted components for the synthesis of assorted nanoparticles (NPs) and nanomaterials is to increase their bioavailability, decrease their potential cytotoxicity, and decrease their preparation costs [6,15]. A wide range of studies has been conducted on using different forms of biowastes, without paying enough attention to the basis of their natural constituents, by only using them as reducing agents and stabilizing agents. However, it is imperative to characterize the functional groups and physicochemical properties of the chemical components of the leaf extracts and biowastes prior to their usage. For instance, by using nitrogen (N)-rich biowastes, the prepared NPs could have the ability to be used as non-viral gene delivery vectors [16]. While deploying mostly linear and non-aromatic chemical compositions of the natural components, the NPs that ensued possess higher stability in some solvents [17]. The underlying task is to uncover how these physical or chemical characteristics are transferred to the final NPs. To answer this question, it should be noted that such transition of these characteristics to the final NPs would be possible only if the final material is not calcined and not subjected to elevated temperatures. In this scenario, chains of these natural constituents are decorated on the NPs surface and lead to limiting some interactions with the cell wall. Many studies have been devoted to CDs [18,19,20,21], but in this study, the necessity of using biowastes for the cost-effective and sustainable production of these materials has been emphasized with reference to their biomedical applications. The presence of cellulose in the biowaste aids in altering the features of the nanomaterials, such as size, morphology, porosity, and dispersion capability. Therefore, biowaste-resulting CDs are discovered to have different uses in energy storage and other advanced technologies.

## 2. Waste to Wealth: Biowaste-Derived CDs with Biomedical Potential

Different precursors and chemicals have been used for fabricating CDs, such as ammonium citrate, ethylene glycol, citric acid, ethylene diamine tetra acetic acid (EDTA), phytic acid, phenylenediamine, thiourea, carbon nanotubes, and graphite, among others [13,14,18]. However, a vast number of green carbon precursors have been utilized for fabricating CDs, such as fruit (e.g., fruit juices and fruit peels), animal and animal-derived materials, vegetables and spices, and waste kitchen materials such as frying oils, waste papers, and plant leaves, among others. The synthesis resources/strategies and optimization techniques/assays, as well as post-synthesis procedures, are vital criteria affecting the morphology/size and properties of CDs [22,23]. Notably, the size of CDs is crucial for understanding their quantum phenomena, which have important effects on biomedical and optoelectronics applications. After the preparation, some complex separation methods are required to obtain monodispersed CDs, including dialysis, chromatography, gel electrophoresis, and ultra-filtration [21,24]. There are two main bottom-up and top-down strategies for the synthesis of CDs with some important disadvantages and advantages. Top-down methods such as arc discharge, laser ablation, and electrochemical oxidation may need complex steps or harsh conditions, and the obtained CDs typically present graphite-like structures with weak fluorescence luminescence (quantum yield is typically less than 10%). On the other side, bottom-up methods such as combustion, pyrolysis, solvothermal, microwave, ultrasonic, and template are relatively easy, cost-effective operations, and they lead to the formation of CDs that are typically amorphous, with relatively high quantum yields [25,26]. Several crucial parameters can affect the properties of CDs, for instance, pH of reaction conditions, temperature, time, and type of precursor materials deployed, along with the amount of carbon present [27,28]. In addition, their properties depend on the heteroatom co-doping of CDs and surface passivation. Notably, the source of CDs can affect their fluorescence property. It was revealed that CDs fabricated from pineapple peels were totally degraded within a few days due to fungal activity, while CDs obtained from cucumber exhibited better stability and no such fungal degradation could be detected [29]. Conversely, CDs prepared from coconut husks with a wide range of functional groups on their surfaces could be used for pH sensing applications [12]. In addition, N-doped CDs prepared from crab shell via a sonochemical-assisted green synthesis technique exhibited low toxicity and high fluorescence quantum yield along with improved diagnostic imaging and an enhanced targeting effect [30]. Overall, the source and type of biowaste used can affect the stability, size, and morphology of the formed CDs and thus affect their functionality, properties, and efficiency [27,31].

**Figure 2:**
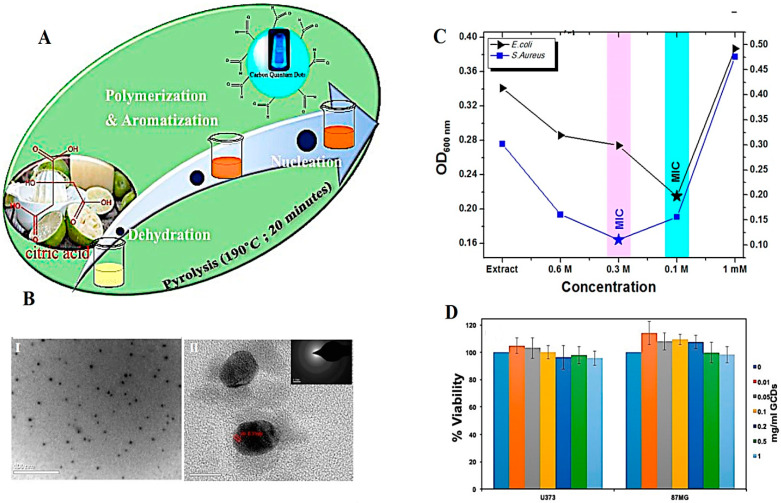
(**A**) The preparative process of CDs using *C. Limetta* waste pulp. (**B**) (**I**) High-resolution transmission electron microscopy (HR-TEM) image (scale bar: 100 nm), (**II**) lattice fringe analysis (scale bar: 10 nm). (**C**) OD_600_ measurements of bacterial cultures incubated with CDs with different mM for analyzing minimum inhibitory concentration (MIC). (**D**) The viability analysis of cells treated with CDs using CKK8 assay. Reproduced with permission from Ref [37]. Copyright 2018 American Chemical Society.

Biowaste resources have been widely utilized as inexpensive and renewable resources with biodegradability, biocompatibility, and availability advantages in the production and functionalization of CDs. However, optimization and feasible studies ought to be addressed for industrial production of biowaste-derived CDs with controllable properties, based on green chemistry principles [32]. In this context, these nanomaterials have been widely explored for biotechnological and biomedical purposes owing to their attractive wavelength-tunable emission, photoluminescence, and other chemico-mechanical properties (Table 1). For instance, hydrophilic N-doped CDs were successfully synthesized as nanoprobes with high biocompatibility for health care and biosensing applications using banana peel biowastes [33]. Metal-doped and hybrid CDs with unique properties have illustrated promising biomedical potentials because of their significant antibacterial, antifungal, antioxidant, and photothermal/photodynamic therapy effects [14]. Peng et al. [34] discussed recent advancements related to CD composites such as metal CDs, multi-component CDs, non-metallic inorganic CDs, and organic CDs with various bio-applications, namely, drug delivery and biosensing/bioimaging [34]. In one study, biowaste-derived CDs/hydroxyapatite nanocomposites synthesized from sugarcane bagasse char precursor via a hydrothermal technique could be deployed as nanocarriers for delivery of acetaminophen, with a loading capacity of ~48.5% [35]. Alternatively, CDs with high chemical stability, low toxicity, and good biocompatibility can be regarded as attractive candidates for antibacterial applications [36]. For instance, CDs were synthesized using *Citrus limetta* waste pulp via a pyrolysis technique (Figure 2). These NPs (~4–7 nm) with quantum yield of 63.3% exhibited suitable antibacterial activity against *Escherichia coli*, and *Staphylococcus aureus*. Such CDs with high optical and structural quality should be further explored for sensing/bioimaging applications [37].

**Figure 3:**
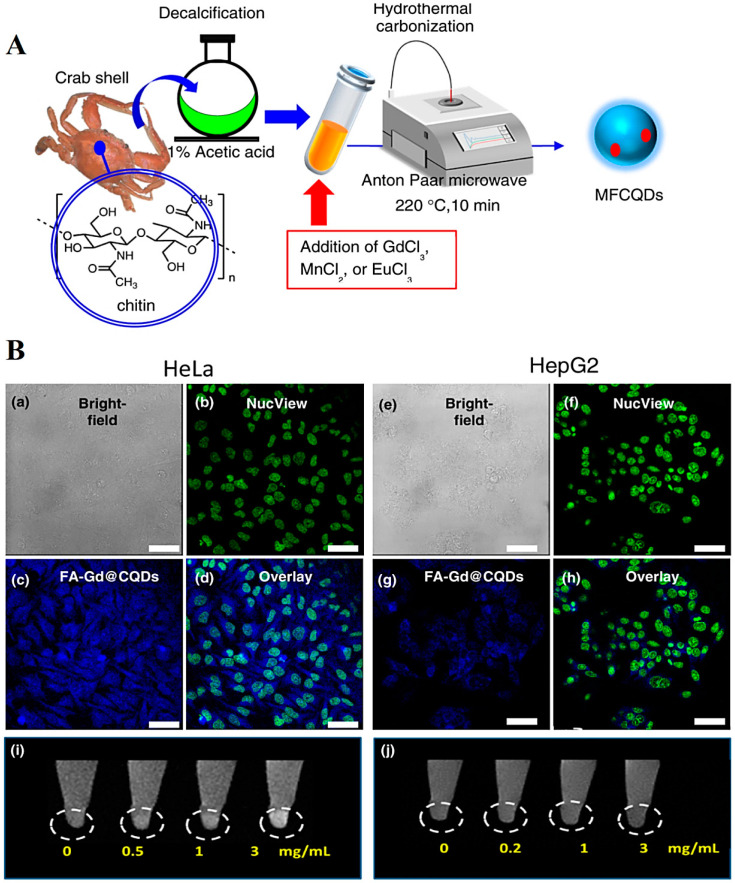
(**A**) The preparative process of magnetofluorescent CQDs through a microwave-assisted hydrothermal technique. (**B**) Fluorescence images of (**a**–**d**) HeLa and (**e**–**h**) HepG2 cells incubated with folic acid (FA)–Gd@CQDs (Scale bars = 40 μm). T_1_-weighted (**i**) HeLa and (**j**) HepG2 cellular magnetic resonance images of FA–Gd@CQDs at different concentrations are illustrated. MFCQDs: magnetofluorescent CQDs. Reproduced with permission from Ref [53]. Copyright 2017 American Chemical Society.

CDs exhibit unique optical features such as up-conversion fluorescence, photoluminescence, and ultraviolet absorption, which make them attractive candidates in designing various sensing platforms with specific diagnostic potentials [45,46]. The strategies for improving the fluorescence properties of CDs have been discussed by Wan et al. [47]. These nanomaterials as emerging photonic nanoagents, in comparison to the conventional fluorophores, exhibited promising applicability in phytomedicine, especially photoacoustic imaging, as well as photodynamic/photothermal therapy of cancers and malignancies [48]. Kitchen wastes such as crown daisy leaves or waste tea residue have been explored for the synthesis of CDs; the resulting CDs can be used for biomedical applications, including drug delivery and bioimaging/biosensing [49,50]. However, their toxicity and biocompatibility issues ought to be systematically addressed [51]. For instance, N-doped CDs synthesized from crown daisy leaves exhibited unique fluorescence quenching property, making them suitable as effective label-free fluorescent probes for sensing applications [52]. To analyze the theranostic potential of biowaste-derived CDs, a one-pot microwave-assisted hydrothermal method was deployed for the synthesis of magnetofluorescent carbon quantum dots (CQDs) from waste crab shell and metal ions, such as Mn^2+^, Gd^3+^, Mn^2+^ and Eu^3+^ (Figure 3) [53]. Accordingly, the designed Gd@CQDs could be applied as T_1_ contrast agents (r1 relaxivity was 4.78 mM^–1^·s^–1^) for bioimaging applications along with targeted anticancer drug delivery. They were further conjugated with folic acid for targeted delivery of doxorubicin to folate receptor-positive HeLa cancer cells, offering promising theranostic agents with cost-effectiveness, high biocompatibility, ease of fabrication, and a reduced environmental impact [53].

**Figure 4:**
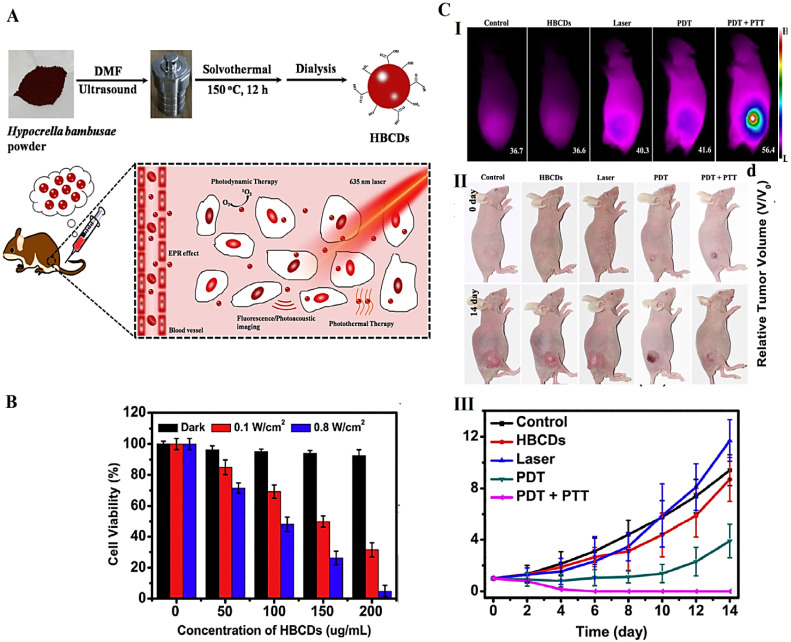
(**A**) The preparative process of CDs derived from *H. bambusae* (HBCDs) with bimodal fluorescence/photoacoustic imaging and synergistic photodynamic/photothermal therapy potentials. (**B**) Cell viability analyses of HeLa cells treated with biowaste-derived CDs. (**C**) Infrared thermal images of mice post various treatments (**I**). Photographs of mice post different treatments (**II**). The growth curves of tumors during various treatments (**III**). DMF: dimethylformamide. Reproduced with permission from Ref [54]. Copyright 2018 Elsevier.

Biowaste-derived CDs can be applied in bioimaging and photodynamic therapy (in vivo) [8]. Jia et al. [54] utilized *Hypocrella bambusae* as a precursor to fabricate CDs by solvothermal method (Figure 4). These CDs exhibited red light emission (maximum peak at 610 nm), low bio-toxicity, suitable water solubility, and broad absorption (350–800 nm); they also could significantly produce ^1^O_2_ (0.38) and heat (27.6%) under 635 nm laser irradiation. Such nanomaterials with unique physicochemical properties have been deployed for synergistic photodynamic and photothermal therapy along with the guidance of bimodal fluorescence/photoacoustic imaging. They display efficient in vivo and in vitro phototherapeutic efficacy upon 635 nm laser irradiation for cancer theranostic applications, thus opening a new window of opportunity for the utilization of natural biomass wastes to generate high-value products with clinical and biomedical potentials [54]. However, specific nanotoxicological evaluations as well as clinical translation studies still need to be addressed for such biowaste-derived nanosystems.

Fresh tender ginger-derived CDs exhibited cellular toxicity to HepG2 when IC_50_ (the half maximal inhibitory concentration) was at a concentration of 350 μg mL^−1^ [55]. In addition, the fabrication of CDs from natural polysaccharides existing in sweet lemon peel via a hydrothermal technique was developed. Sweet lemon peel is food waste; these peels were applied as biowastes to synthesize CDs. These CDs were then utilized for green synthesis of silver (Ag) NPs (~5–16 nm) with suitable antibacterial effects against *Escherichia coli* (~80% inhibition potential). The CD@AgNPs exhibited a dose-dependent assay’s bigger anticancer potency against MCF 7 cell lines. It was detected that the anticancer effects of these nanomaterials against MCF 7 was intensely associated with the synthesis of reactive oxygen species (ROS), causing apoptosis. The importance of CDs is due to their prominent optical properties, such as the excitation of multi-color fluorescence, which can transform them into a bioimaging probe to diagnose diseases in the future [56]. CDs also show the efficacy of intracellular fluorescence cells following A549 lung cancer cells [57].

CDs have shown great potential for tissue engineering and regenerative medicine [58,59,60]. For instance, chitosan–CD nanocomposites were constructed for enhancing wound healing [61]. In another study, eggshell-derived calcium phosphate/CD nanofibrous scaffolds were constructed for bone tissue engineering. These scaffolds exhibited improved alkaline phosphatase activity and cell proliferation rate, thus serving as potential candidates for bone tissue regeneration [62]. Furthermore, bone morphogenetic protein-2 (BMP-2)-conjugated CDs were embedded in gelatin-elastin-hyaluronic acid hydrogel scaffolds for bone tissue engineering applications; the BMP-2-CDs could exhibit sustained released from the scaffolds for up to 21 days. In vitro studies illustrated the intercellular uptake of BMP-2-CDs along with the improved biological properties and pro-osteogenic effect [63]. CDs have also been conjugated with vascular endothelial growth factor for protein tracking in angiogenic therapy and tissue engineering applications and deployed for in vitro imaging of human umbilical vein endothelial cells [64].

Solutions of CDs have been utilized for the surface modification of polycaprolactone scaffolds to increase their hydrophilicity [65], where an improvement in cell proliferation and attachment to the modified scaffolds could be obtained. These CD-based scaffolds exhibited good biocompatibility, surface roughness, pore structure, and hydrophilicity, making them suitable for tissue engineering applications [65]. In addition, citric acid-based CDs were developed for labeling and tracking of rat bone marrow mesenchymal stem cells [66]. These extremely fluorescent probes provided labeling of rat bone marrow mesenchymal stem cells by internalization without affecting cell viability or stimulating apoptosis when the concentration was lower than 50 μg mL^−1^. Notably, CDs could facilitate osteogenic differentiation of rat bone marrow mesenchymal stem cells with high efficiency through the stimulation of osteogenic transcription and enhancement of matrix mineralization [66]. For bone regeneration and fracture healing, N-doped CDs conjugated with hydroxyapatite nanomaterials were constructed with cell imaging capability, improved alkaline phosphatase activity, mineralization, and expression of the osteogenic genes in osteoblast cells. They could highly improve zebrafish bone regeneration and mineral density compared to hydroxyapatite nanomaterials [67].

## 3. Challenges and Future Perspectives

In recent years, much consideration has been paid to the application of natural resources in the synthesis of CDs via a hydrothermal carbonization technique due to the need for a nominal experimental setup. CDs synthesized through the use of some natural sources such as milk and fruit skins often have high quantum yield. Although CDs produced through different paths or precursors exhibited other optical features to different wavelengths, the type of source and synthesis method are very important criteria [68,69]. Semiconductor quantum dots are favored candidates with excellent biomedical potentials, particularly probable usage as optical bioimaging agents. The study of photoluminescence properties and surface chemical conformation of CG (bovine gelatin) and CA (PHM3 algae) nanodots proposed that the surface chemical structure could significantly change the surface conditions, which could directly affect their photoluminescence features. CG nanodots were also applied to imaging cells with higher imaging sensitivity than semiconductor quantum dots, with possible toxicity. The in vitro results illustrated the effective anticancer effects of these nanodots, offering them as potential alternatives for imaging and biomedical purposes [70].

Additional explorations are warranted to fabricate CDs with enough excitation and emission in red/near-infrared regions, which drastically affect their merits and practical functions in biomedical therapies/assays [71]. Nevertheless, the lack of a comprehensive guideline for optimizing and industrializing the production of CDs, especially from biowaste resources and based on green chemistry approaches, is felt more than ever before. The future directions of CDs with biomedical potentials are yet to be deliberated, focusing on the challenges encountered in industrial development regarding the impact of raw materials and the effect of temperature, reaction time, pH value, heteroatom co-doping, and surface passivation on the structures and properties (e.g., optical, structural, cytotoxicity, biocompatibility, tunability, stability, and catalytic activity properties) of CDs [72]. The improvement of their surface passivation, the control of emission color and color intensity, and the stabilization of their optical features are crucial aspects that need to be considered [11]. Notably, the photoluminescence, chemiluminescence, and absorption mechanisms, in addition to the associated photophysical procedures of photothermal conversion and generation of ROS, ought to be carefully deliberated [46].

## 4. Conclusions and Future Outlooks

Biowaste-derived CDs with high biocompatibility and biodegradability properties have recently garnered immense attention from researchers; biowastes can be deemed as natural, low-cost, biorenewable, economical, and green resources for the preparation of multifunctional CDs deployable in the field of sensing/imaging, optoelectronics, photocatalysis, drug/gene delivery, tissue engineering, regenerative medicine, and cancer theranostics. The future explorations in imaging and sensing ought to be focused on additional improvement of selectivity/sensitivity and stability of CD-based nanosystems; the interference reduction from background and auto-fluorescence from biological analyses are essential. Biowaste-derived CDs can provide an alternative treatment for anticancer therapy, as well as malignant and chronic diseases. This creates a much-needed paradigm shift for additional clinical studies in the field of fundamental and applied green and natural product-based nanotechnology, with the merits of improved bioavailability, high drug loading capacity, multifunctionality, inexpensive materials, and biosafety.

CD-based nanosystems, with their fascinating prolonged/targeted drug release behavior, labeling features, and high efficacy-to-toxicity ratio, can be judged as smart materials for next-generation theranostic applications after surface functionalization/modification using suitable biocompatible/bioactive agents. The control of size/morphology, chemical doping, and functionalization can help to improve the properties of CDs (e.g., optical properties) for specific biomedical purposes. Despite the progress achieved, important challenging issues regarding the absorption/emission wavelength, up-conversion emission/multiphoton fluorescence mechanisms, and phosphorescence of these CDs still linger, which need to be addressed for deriving the maximum benefits from biowaste-derived CDs. Environmentally benign fabrication of CDs that consider the important factors affecting the sizes/morphologies and properties of the final product is crucial.

## Figures and Tables

**Table 1:** Some selected examples of biowaste-derived CDs with their fascinating biomedical potential.

Sources	Synthesis Methods	Biomedical Potentials	Size (nm)	Refs.
*Citrus limetta* waste pulp	Pyrolysis method	Fe(III) ions sensing, bactericidal performance, and bioimaging	4–7	[37]
Jackfruit peel and tamarind peel precursors	Hydrothermal synthesis	Anticancer and antitumor activity	6.4	[38]
*Actinidia deliciosa* (kiwi) peels	Hydrothermal-carbonization method	Cell labeling agents for mesenchymal stem cells and breast/thyroid cancerous cells; in vitro imaging	5	[39]
Sugarcane bagasse char	Hydrothermal synthesis	Drug delivery of acetaminophen	7.5	[35]
Sugarcane baggage	Hydrothermal synthesis	Bio-imaging/bio-labeling applications	2–8	[40]
Waste tea leaves; peanut shells	Hydrothermal synthesis	Biosensing; biomarkers	<10	[41]
Crab shells	Sonochemical technique	Cell imaging; theranostic applications	<10	[30]
Silkworm cocoon	Pyrolysis method	Anti- inflammatory potentials	2.26–9.35	[42]
Expired passion fruit shells	Hydrothermal synthesis	Imaging; fluorescent probe	<5	[43]
*Allium sativum* peel	Oxidative pyrolysis technique	Biosensing/cell labeling; biomarker detection (in vitro)	<10	[44]

## Data Availability

Not applicable.
